# Systematic Evaluation of Pharmacokinetic Models for Model-Informed Precision Dosing of Meropenem in Critically Ill Patients Undergoing Continuous Renal Replacement Therapy

**DOI:** 10.1128/aac.00104-23

**Published:** 2023-05-01

**Authors:** Lea Marie Schatz, Alexander Brinkmann, Anka Röhr, Otto Frey, Sebastian Greppmair, Ferdinand Weinelt, Michael Zoller, Christina Scharf, Georg Hempel, Uwe Liebchen

**Affiliations:** a Department of Pharmaceutical and Medical Chemistry, Clinical Pharmacy, University of Muenster, Muenster, Germany; b Department of Anaesthesiology and Intensive Care Medicine, General Hospital of Heidenheim, Heidenheim, Germany; c Department of Pharmacy, General Hospital of Heidenheim, Heidenheim, Germany; d Department of Clinical Pharmacy and Biochemistry, Institute of Pharmacy, Freie Universitaet Berlin, Berlin, Germany; e Department of Anesthesiology, University Hospital, LMU Munich, Munich, Germany

**Keywords:** meropenem, continuous renal replacement therapy, continuous kidney replacement therapy, critically ill, model-informed precision dosing, population pharmacokinetic, sepsis, Bayesian forecasting, therapeutic drug monitoring

## Abstract

The altered pharmacokinetics of renally cleared drugs such as meropenem in critically ill patients receiving continuous renal replacement therapy (CRRT) might impact target attainment. Model-informed precision dosing (MIPD) is applied to individualize meropenem dosing. However, most population pharmacokinetic (PopPK) models developed to date have not yet been evaluated for MIPD. Eight PopPK models based on adult CRRT patients were identified in a systematic literature research and encoded in NONMEM 7.4. A data set of 73 CRRT patients from two different study centers was used to evaluate the predictive performance of the models using simulation and prediction-based diagnostics for i) *a priori* dosing based on patient characteristics only and ii) Bayesian dosing by including the first measured trough concentration. Median prediction error (MPE) for accuracy within |20%| (95% confidence intervals including zero) and median absolute prediction error (MAPE) for precision ≤ 30% were considered clinically acceptable. For *a priori* dosing, most models (*n* = 5) showed accuracy and precision MPE within |20%| and MAPE <35%. The integration of the first measured meropenem concentration improved the predictive performance of all models (median MAPE decreased from 35.4 to 25.0%; median MPE decreased from 21.8 to 4.6%). The best predictive performance for intermittent infusion was observed for the O’Jeanson model, including residual diuresis as covariate (*a priori* and Bayesian dosing MPE within |2%|, MAPE <30%). Our study revealed the O′Jeanson model as the best-predicting model for intermittent infusion. However, most of the selected PopPK models are suitable for MIPD in CRRT patients when one therapeutic drug monitoring sample is available.

## INTRODUCTION

Rapid attainment of effective antibiotic concentrations reduces mortality in septic patients (1, 2). However, optimal dosing of critically ill patients is challenging, as they are characterized by constantly changing pharmacokinetics due to pathophysiologic alterations (e.g., an increasing volume of distribution during sepsis, altered renal clearance) (3). In addition, the use of supportive extracorporeal therapies such as extracorporeal membrane oxygenation (ECMO) or continuous renal replacement therapy (CRRT) further hampers precision dosing (4).

Especially, the status of the renal function is important for dosing of meropenem since it is predominantly (70%) renally cleared (5). If CRRT is required, total meropenem clearance is significantly reduced, but the proportion of extracorporeal removal is high due to its physicochemical properties such as low molecular weight and low plasma protein binding (6). Moreover, the type of CRRT or CRRT intensity in terms of flow rate could possibly have an impact on the extracorporeal clearance of meropenem (7). Furthermore, it might be relevant to take residual diuresis of the patient into account (8).

All these influencing factors have an individual impact on the pharmacokinetics of meropenem and the “one dose fits all” dosing paradigm is not sufficient. Therefore, therapeutic drug monitoring (TDM) is recommended to avoid under- and overdosing (9, 10). However, dose adjustments can only be made after treatment has started and TDM for meropenem in hospitals is rarely available (9, 11). The use of population pharmacokinetic (PopPK) models provides the opportunity to determine individualized dosing before treatment is initiated (12).

This project aimed to identify suitable PopPK models from the literature for the use of model-informed precision dosing (MIPD) of meropenem in CRRT patients, i.e., i) for *a priori* dosing based on patient characteristics only and ii) for Bayesian dosing by integrating one measured plasma concentrations into dosing decisions.

## RESULTS

### PopPK models for CRRT patients.

The populations of the eight selected models varied in size of critically ill patients (*n* = 10 to 101). The number of CRRT patients was even lower (*n* = 6 to 49). Besides, all eight models were heterogeneously structured and contained different covariates (see Table S5 in the supplemental material). All study designs were based on intermittent infusion of meropenem. Most PopPK models were two-compartment models (*n* = 6) that did not include a CRRT parameter such as flow rate or dialysis type as a covariate (*n* = 7). Only the model by Burger et al. 2018 (24) included total flow rate as a structural covariate on clearance. Further covariates for clearance were described by either residual diuresis in two models (O’Jeanson et al. [8] and Ulldemolins et al. [25]) or eGFR in two models (Niibe et al. 2022 [26] and Hanberg et al. [27]). Nevertheless, most models (*n* = 6) included only one significant covariate.

### Model evaluation for *a priori* dosing.

**FIG 1:**
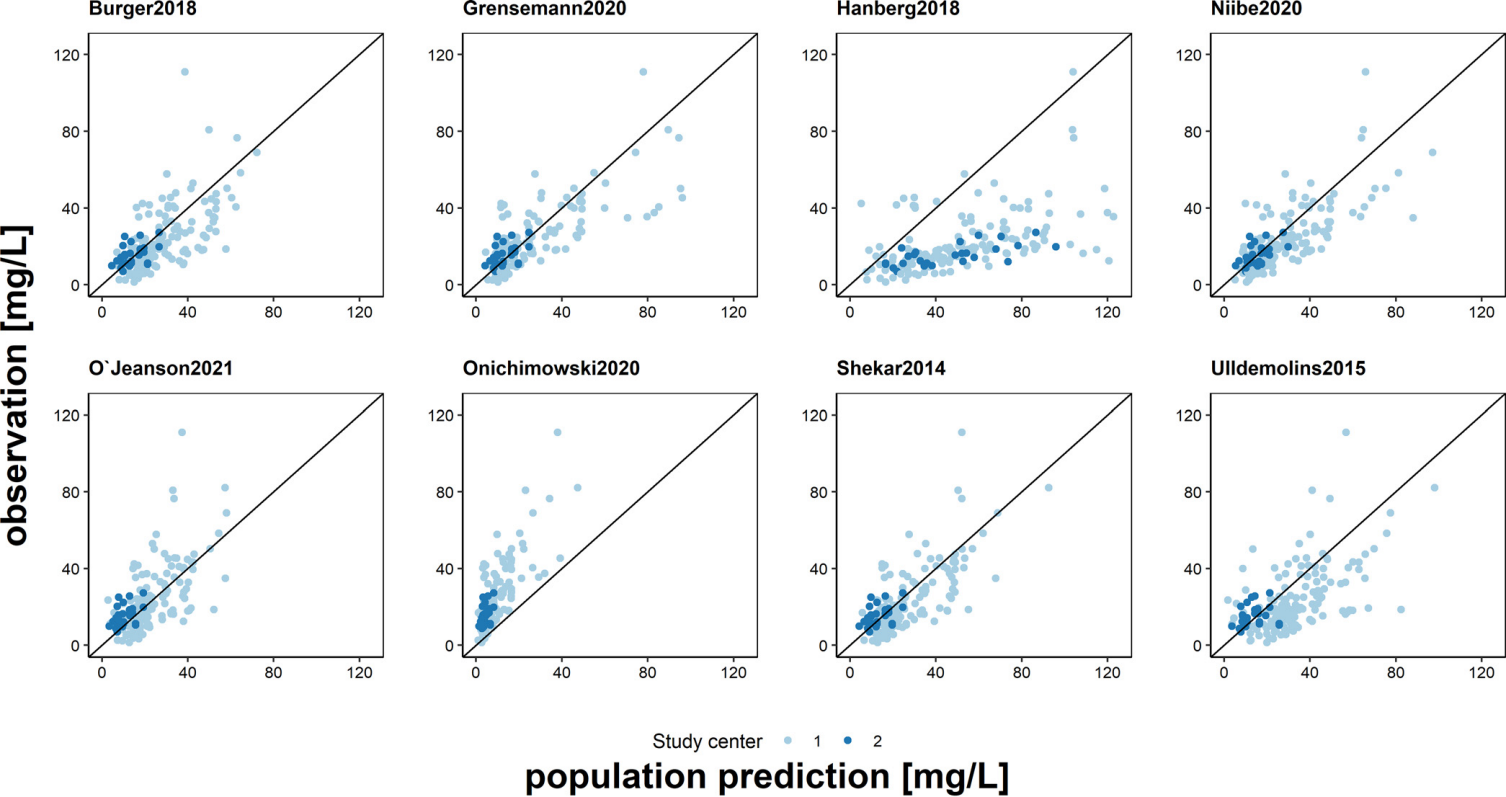
Goodness-of-fit plot: population predictions of the investigated models versus observations of all measured meropenem plasma concentrations. Black line: line of identity.

Based on the total data set the selected models showed a high discrepancy of their predictive performance for *a priori* dosing with the median prediction error (MPE) varying from −98.7 to 94.9% and the median absolute prediction error (MAPE) from 26.7 to 98.7%. The model by O’Jeanson et al. 2021 (8) met the predefined criteria for clinical acceptance (*a priori* prediction for accuracy [MPE = 0.13%; CI: –7.1 to 9.9%] and precision [MAPE = 29.4%]) (all results are shown in Table S6). The overall model correctness for this PopPK model was confirmed by statistical tests of normality for the calculated normalized prediction distribution errors (NPDEs) (O’Jeanson et al. 2021 [8]; difference of the mean, 0.12 [*P* = 0.2]; Fisher variance, 0.97 [*P* = 1]); however, the prediction-corrected visual-predictive checks (pcVPCs) showed a misfit for continuous infusion (Fig. S7 and S9, Table S8). The models by Shekar et al. 2014 (28), Burger et al. 2018 (24), Grensemann et al. 2020 (29), and Niibe et al. 2020 (26) showed no bias in the goodness-of-fit (GOF) plots (Fig. 1) and an accuracy and precision of MPE |20%| and MAPE of <35%, respectively, whereas the models by Onichimowski et al. 2020 (30) and Hanberg et al. 2018 (27) showed a strong visual bias in the GOF plots, a MAPE of >90% and a misfit in the pcVPCs (Fig. 1, Fig. S9). Besides the O’Jeanson model (8), the model correctness could only be confirmed for the model by Shekar et al. 2014 (28) (difference of the mean −0.11 [*P* = 0.25]; Fisher variance, 0.77 [*P* = 0.05]), which showed a small bias in the MPE (MPE = 12.7% [CI: 7.6 to 18%]) (Table S6 and S8, Fig. S7).

When stratified by center, the results were slightly different, but MPE was mostly still in |25%| and MAPE <35% for well-predicting models. However, a center-dependent trend of MPE in model predictions could be identified for these models. For center 1, the models mostly overestimated the measured concentration (MPE > 0%), whereas for center 2, the models mostly underestimated the measured concentration (MPE < 0%). The 95% confidence intervals (CI) often intersected zero still for at least one center.

Significant differences in model predictions between centers were seen in three models. The model by O’Jeanson et al. 2021 (8) was more accurate for center 1 compared to center 2, whereas the models by Burger et al. 2018 (24) and Shekar et al. 2014 (28) provided more accurate predictions for center 2 than for center 1. The width of the 95% CI for center 1 as well as in pcVPCs for intermittent infusion was significantly smaller than center 2 and continuous infusion (Table S6, Fig. S9).

### Model evaluation for Bayesian dosing.

**FIG 2:**
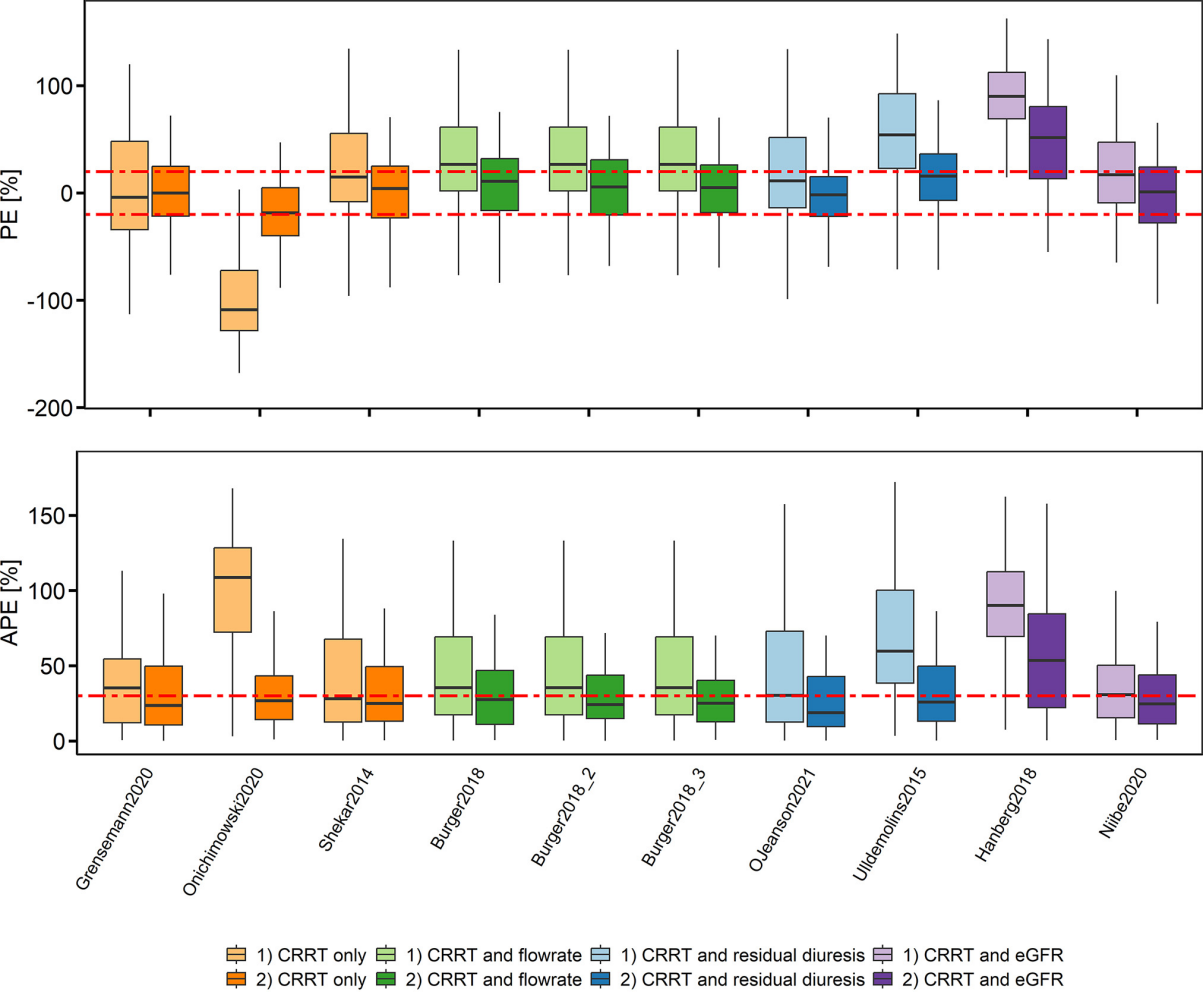
Relative prediction error (PE) and the relative absolute prediction error (APE) of the predicted versus the observed meropenem concentrations for the trough concentration of the second dosing occasion for i) *a priori* prediction using the patient covariates only, and ii) Bayesian forecasting prediction using the last measured concentration of the first dosing occasion. The models were grouped according to the included covariates on clearance. Box plots represent the 25^th^, 50^th^, and 75^th^ percentiles; the whiskers cover the 1.5-fold interquartile range; red dashed line represents clinically acceptable values ±20% for PE and 30% for APE. Burger et al. 2018 (25) is presented with three different residual errors published.

By including the trough concentration of the first occasion the median MAPE of all models for the second occasion improved from 35.4 (*a priori*) to 25.0% (Bayesian) (median MPE of all models = 21.8 to 4.6%). Most models showed acceptable accuracy and precision for clinical use except the models by Hanberg et al. 2018 (27), Onichimowski et al. 2020 (30), and Ulldemolins et al. 2015 (25) (Fig. 2, Table S10). The superiority of a covariate on clearance could not be identified when comparing the predictive performance of the models (Fig. 2).

## DISCUSSION

The evaluated models from the literature were structured heterogeneously and based on small patient cohorts (n_patients_ undergoing CRRT ≤ 46), which can be explained by the special populations investigated. The patients were not only critically ill, which *per se* leads to rapidly changing pharmacokinetics, but they also required CRRT to replace their renal function. The small patient populations might explain the large differences in predictive performance of the models and the few numbers of significant covariates included. Our external data set, consisting of 73 patients from two different centers, was larger than the model populations.

Based on our data set, the model by O’Jeanson et al. 2021 (8) (included residual diuresis as covariate on clearance) met our predefined criteria to be considered clinically acceptable for *a priori* dosing. The model correctness was confirmed by NPDEs for the entire data set and by pcVPCs for intermittent infusion. Therefore, this model can be used for PopPK model-guided dosing in CRRT patients receiving meropenem as intermittent infusion either for initial dose calculation or for hospitals which do not have the possibility to perform TDM. Since meropenem is not regularly monitored (solely in 22 to 26% of the hospitals [9, 11]), the general model evaluation based only on the patients’ characteristics is especially valuable for hospitals lacking TDM facilities.

The models by Shekar et al. 2014 (28), Grensemann et al. 2020 (29), Niibe et al. 2020 (26), and Burger et al. 2018 (24) showed almost acceptable model performance. In contrast to physiological considerations, most previously published models did not identify the type of CRRT or CRRT intensity in terms of flow rate as a significant covariate. Only the model by Burger et al. 2018 (24) contained flow rate as structural covariate. However, this model was more accurate in center 2, where the flow rate was 2,000 mL/h for all patients. In center 1, flow rates up to 5,600 mL/min were used in few patients. Therefore, the model may not predict so well for these values, since this range was presumably not covered by the model population (flow rates were unfortunately not published for all patients in Burger et al. 2018 [24]).

The poorer performance for center 2 compared to center 1 using the model by O’Jeanson et al. 2021 (8) may be explained by the fact that residual diuresis was included in the model as a continuous covariate. In the data set of center 2, residual diuresis was only represented categorically. Nevertheless, the residual diuresis is a plausible covariate, since meropenem is eliminated renally. Therefore, the model performance might be underestimated for center 2. In addition, patients of our external data had significantly lower residual diuresis (median of 0 mL/24 h) than patients in the model development population (median of 845 mL/24 h by the model of O’Jeanson et al. 2021 [8]). The trend observed between the two centers in terms of accuracy can possibly be explained by different patient population. Center 1 is the university hospital of a large city in contrast to the hospital of center 2. Therefore, the patients of center 1 are probably more seriously ill. It is also noticeable that the patients in center 1 are comparatively younger than in center 2 (median age 56 years versus 76 years, respectively).

Although the well-predicting models differed in structure and included covariates, the parameter values for the clearance with the median patients characteristics for covariates of our data set were comparable (Burger et al. 2018 [24]: 4.7 L/h [with median total flow rate of 2,000 mL/h], Grensemann et al. 2020 [29]: 5.06 L/h, O’Jeanson et al. 2021 [8]: 5.78 L/h [with no residual diuresis], Shekar et al. 2014 [28]: 5.1 L/h, Niibe et al. 2020 [26]: 4.4 L/h [with median eGFR (Cockcroft-Gault) of 31 mL/min/1.73 m^2^]. However, the models with a clear bias either overestimated or underestimated the clearance in our data set (Onichimowski et al. 2020 [30]: 15 L/h, Hanberg et al. 2018 [27]: 1.9 L/h [with median eGFR (CKD-EPI) of 41 mL/min], respectively).

Unsurprisingly, the inclusion of a TDM sample significantly improved the predictive performance of the models. Therefore, performing a TDM during meropenem therapy is recommendable even if a PopPK model-based dosing approach is used. By including a TDM sample, the unexplained variability described in the models can be better quantified for an individual patient. This can individualize the prediction for the patient and further improve it alongside the covariates. Since all these models were developed to describe the PK in a specific population and not with the intention of being used in MIPD, a “fit-for-purpose” evaluation for MIPD is needed.

Although not all possible patients are included in our data set, because of the large number of patients from two centers, transferability to other centers can be assumed for patients with intermittent infusion with the same range of covariates values. However, the O’Jeanson model (8) might be only the best for the current data set since the results of the well-predicting models are very similar.

Some limitations of the present analysis should be mentioned. First, even if our study population was larger than the model development populations, it still might be biased due to the limited number and specifics of the two centers. Thus, our results may not be generalizable. Although it was not shown in our analysis, the type of CRRT, ECMO, or CRRT intensity could potentially still have an impact on clearance. For example, the model by Hanberg et al. 2018 (27) had a clear bias which was developed specifically for ECMO patients. Particularly, for continuous infusion, the number of patients (*n* = 17) was very small, resulting in a large width of the 95% CI in the statistical analysis for center 2 and for continuous infusion in the stratified pcVPCs. For this reason, our recommendations refer primarily to intermittent administration. Therefore, further multicenter evaluation studies might be valuable, especially if they include more patients with continuous infusion, where our study could be used as a blueprint. Second, we included only one TDM sample to simulate a real-world scenario. Further improvements might be possible if more than one sample were included. However, it was shown by Broeker et al. 2019 that the most recent concentration improves the predictive performance the most (31). Third, we evaluated only a single model approach, a multiple-model approach might be beneficial to handle larger differences in pharmacokinetics.

In conclusion, most of the selected PopPK models are suitable for MIPD in CRRT patients when one sample is included. Based on our data set, the model by O’Jeanson et al. 2021 (8) is the best-fitting PopPK model among the selected models for both *a priori* dosing and Bayesian dosing for intermittent infusion. The estimated clearance value is consistent with other well-predicting models. Including residual diuresis as a covariate is physiologically plausible. Therefore, we recommend implementing a well-predicting model, e.g., O’Jeanson et al. 2021 (8) in a MIPD software tool to make it clinically usable without extensive pharmacometric experience and to further evaluate the integration of different covariates on clearance in a prospective setting.

## MATERIALS AND METHODS

### Literature research and model selection.

In February 2022, a literature search was conducted in Pubmed based on “meropenem,” “CRRT,” and “population pharmacokinetic.” The detailed screening process is described in Table S1 and Fig. S2 and resulted in eight suitable models.

### Clinical data.

Clinical data were collected at two different study centers (center 1: University Hospital of Munich, LMU; center 2: Klinikum Heidenheim, both Germany). The data set from center 1 was composed of three previously conducted studies (registered under clinicaltrials.gov [NCT01793012, NCT03985605] and Institutional Review Board of LMU [registration numbers 428-12, 18-578]). For details regarding the studies, please refer to Ehmann et al. 2019 (13), Scharf et al. 2020 (14), and Liebchen et al. 2021 (15), respectively. Center 2 conducted a retrospective observational study including data from 2014 to 2019. Ethical approval was obtained by the Ethics Committee of the University of Ulm, Germany (project number 137/19).

In the evaluation only patients of these studies with at least 24 h on CRRT after meropenem admission and the availability of at least two measured plasma concentrations in two different occasions were included. An occasion was defined as the time interval including at least one measured plasma concentration between two dosing events or all dosing events until the next plasma concentration was measured. Patients from both centers were treated with meropenem according to the responsible physicians. In center 1, most patients were treated with intermittent infusions, whereas all patients from center 2 received a continuous infusion. Meropenem concentrations were analyzed independently according to previously published methods (center 1: liquid chromatography with tandem mass spectrometry [16, 17]; center 2: high performance liquid chromatography with UV detection [18–20]). For center 2, the residual diuresis was classified into three categories: 1 > 1,000 mL/24 h; 2 = 500 to 1,000 mL/24 h; and 3 < 500 mL/2 h. For the evaluation the values were set to the lower limit (1 = 1,000 mL/24 h, 2 = 500 mL/24 h, 3 = 0 mL/24 h). When covariates were not provided (<11% for both centers), the median of the model population was imputed.

The total data set consisted of 73 patients with 193 plasma concentrations. The patients’ characteristics are summarized in Table 1 and Table S3.

**TABLE 1:** Patient characteristics

	Study center 1	Study center 2	Total
Characteristics^*a*^	*(n = *60)	(*n* = 13)	(*n* = 73)
Sex			
Male	43 (72%)	10 (77%)	53 (73%)
Female	17 (28%)	3 (23%)	20 (27%)
Age (yrs)			
Median (range)	56 (19–87)	76 (55–89)	60 (19–89)
Wt (kg)			
Median (range)	77 (48–140)	85 (55–120)	77 (48–140)
Ht (cm)			
Median (range)	170 (150–190)	170 (160–190)	170 (150–190)
Serum albumin (g/L)			
Median (range)	25 (17–39)	18 (8.0–28)	23 (8.0–39)
Serum creatinine (mg/dL)			
Median (range)	2.0 (0.60–6.1)	2.3 (0.90–8.6)	2.1 (0.60–8.6)
eGFR(Cockcroft-Gault) (mL/min)			
Median (range)	47 (18–180)	36 (5.2–62)	44 (5.2–180)
eGFR(CKD-Epi) (mL/min/1.73m²)			
Median (range)	36 (11–120)	26 (5.2–84)	32 (5.2–120)
Dialysis type			
CVVH	43 (72%)	0 (0%)	43 (59%)
CVVHD	10 (17%)	13 (100%)	23 (32%)
CVVHDF	7 (12%)	0 (0%)	7 (10%)
Total flow rate (mL/h)			
Median (range)	2,000 (1,500–5,700)	2,000 (2,000–2,000)	2,000 (1,500–5,700)
Residual diuresis (mL/24 h)			
Median (range)	0 (0–1,500)	1,000 (0–1,000)	0 (0–1,500)
Dosing strategy			
N patients (II/CI)	56/4	13	56/17
Sampling strategy			
No. samples (total)	167	26	193
No. patients (>2 samples)	11	0	11

a N, Number of patients; Total flow rate, sum of dialysate flow and replacement fluid flow; eGFR, estimated glomerular filtration rate; CKD-Epi, chronic kidney disease epidemiology collaboration; CVVH, continuous veno-venous filtration; CVVHD, continuous veno-venous hemodialysis; CVVHD, continuous veno-venous hemodiafiltration; II, intermittent infusion; CI, continuous infusion.

### Model evaluation strategy.

All models were encoded in NONMEM 7.4 (Icon Development Solutions, Hanover, MD, USA) based on the provided information in the publication (find encoded models in Supplemental File S4). The statistical and graphical evaluation were performed using R version 4.0.3.

**FIG 3:**
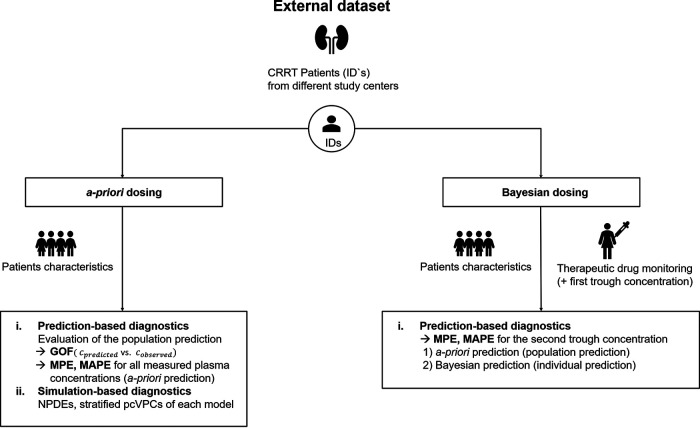
Model evaluation workflow for model-informed precision dosing. CRRT, continuous renal replacement therapy; GOF, goodness-of-fit-plots; MPE, median prediction error; MAPE, median absolute prediction error; NPDE, normalized prediction distribution error; pcVPC, prediction-corrected visual-predictive-check.

The workflow for the evaluation is presented in Fig. 3. At the start of meropenem treatment, only patients’ characteristics (but no TDM results) are available for calculating the initial dose. Therefore, the model evaluation for *a priori* dosing was based on population predictions of the models, including only the covariates of the patients but no TDM measurement (*a priori* prediction for all measured concentrations). To investigate the improvements that could be achieved by including TDM measurements during the treatment course, a Bayesian forecasting strategy (Bayesian dosing) was evaluated: the predictive performance of the models for the trough concentration of the second occasion was evaluated i) only based on patients’ characteristics (*a priori* prediction) and ii) by including the measured trough concentration of the first dosing occasion and using Bayesian forecasting to predict the second trough concentration (Bayesian prediction).

Median prediction errors (MPE) were calculated to assess the accuracy and median absolute prediction errors (MAPE) for the precision according to the following equations:

**PE for a single time point.**

(1) $$PE_{i,j}=\frac{c_{\mathrm{pred},i,j}\;-\;c_{\mathrm{obs},i,j}}{(c_{\mathrm{pred},i,j}\;+\;c_{\mathrm{obs},i,j})/2}$$

where *c_pred,i,j_* is the predicted and *c_obs,i,j_* the observed concentration of the *i*th individual with the *j*th measured plasma concentrations.

**MPE for model accuracy.**

(2) $$\mathrm{MPE}\;\left[\text{\%}\right]=\text{median}(\{\text{r}{\mathrm{PE}}_{1,1},\ldots ,\text{r}PE_{\mathrm{i,j}}\})\text{ × }100$$

**MAPE for model precision.**

(3) $$\mathrm{MAPE}\;\left[\text{\%}\right]=\text{median}\;(\{|\text{r}{\mathrm{PE}}_{1,1}|,|\ldots |,|\text{r}PE_{\mathrm{i,j}}|\})\text{ × }100$$

MPE between −20 and 20% with the 95% CI including zero and a MAPE ≤ 30% were considered acceptable criteria for accuracy and precision, respectively (21–23). In addition, NDPEs and prediction-corrected pcVPCs stratified by different administration routes were used as simulation-based tools to evaluate model prediction performance, including variability.
